# Tai Chi Chuan teaching on alleviating mental fatigue among college students: insights from ERPs

**DOI:** 10.3389/fpsyg.2025.1561888

**Published:** 2025-04-09

**Authors:** Ruimin Ji, Jiayu Li

**Affiliations:** ^1^Department of Physical Education, Sports Institute of Chengdu University of Technology, Chengdu University of Technology, Chengdu, Sichuan, China; ^2^Wushu Department, Wushu College of Henan University, Henan University, Kaifeng, Henan, China

**Keywords:** Tai Chi Chuan teaching, college students, mental fatigue, EEG, event-related potentials

## Abstract

**Background:**

Mental fatigue is a prevalent issue, especially among college students, which significantly impacts learning efficiency. Existing interventions like caffeine use have side-effects, highlighting the need for alternative approaches. Tai Chi Chuan (TCC), a traditional Chinese mind -body exercise, shows promise in alleviating mental fatigue, yet its neural mechanisms on alleviating mental fatigue, particularly from the perspective of inhibitory control, remain unclear.

**Objectives:**

This study aimed to fill this gap by investigating the effects of TCC on mental fatigue and cognitive function in college students using ERP measures based on the Go/NoGo task.

**Methods:**

Fifty-two healthy college students were randomly assigned to the TCC group (*n* = 18), aerobic exercise (AE) group (*n* = 17), or control (CON) group (*n* = 17). Mental fatigue was induced via the Stroop task, followed by a 20-min intervention of TCC practice, ergometer exercise, or sitting rest. Reaction time (RT), Visual Analog Scale (VAS) scores, and EEG data were collected.

**Results:**

The data showed that after the Stroop task, all groups exhibited signs of mental fatigue, with increased VAS scores and RTs. However, following the 20-min intervention, the TCC group demonstrated more significant improvements in VAS scores, RTs, and ERP components (such as increased NoGo-P3 and Go-P3 amplitudes, shortened Go-P3 latencies, and decreased NoGo-N2 amplitudes) compared to the AE and CON groups.

**Conclusion:**

These findings indicate that practicing TCC can effectively alleviate mental fatigue and facilitate cognitive recovery in college students after sustained cognitive tasks. This study provides new insights into the neural mechanisms of TCC’s beneficial effects on alleviating mental fatigue.

## Introduction

Recently, mental health issues have received growing attention (Lee et al., 2024; Tuan et al., 2024). Mental fatigue is a complex psychological state characterized by a subjective sense of exhaustion and a decline in cognitive performance, which can significantly impair an individual’s daily functioning (Filtness and Naweed, 2017). It is not merely a transient state of tiredness but a condition that can significantly impact an individual’s ability to perform tasks efficiently, make sound decisions, and maintain a high learning efficiency (Filtness and Naweed, 2017; Peng et al., 2021).

Previous research has explored various strategies to alleviate mental fatigue. For instance, the use of bioactive substances like caffeine has been investigated, yet it comes with potential side-effects such as abnormal heart rhythms and insomnia (Azevedo et al., 2016; Nawrot et al., 2003). Other alternative approaches, including smelling special odors (Kato et al., 2012) and listening to relaxing music (Guo et al., 2015; Gao et al., 2023), have also been studied, but their effectiveness remains inconsistent. Physical exercise, on the other hand, has shown promise in enhancing cognitive performance. Beneficial effect of physical exercise has been largely reported for cognitive performance (Oberste et al., 2019). A functional magnetic resonance imaging investigation demonstrated that engaging in a 10-min session of light-intensity cycling on an ergometer could effectively enhance cognitive function. This beneficial outcome of physical activity was associated with an elevated state of alertness (Byun et al., 2014). Therefore, acute physical exercise could also be a promising and effective strategy to counteract mental fatigue. In particular, the practice of Tai Chi Chuan (TCC), a traditional Chinese mind–body exercise, has attracted increasing attention for its potential positive impacts on mental health and cognitive functions. Existing studies have demonstrated that TCC can enhance neural plasticity, improve mood states, reduce stress, and contribute to better sleep quality (Caldwell et al., 2016; Shen et al., 2021; Yao et al., 2021; Yu et al., 2018). However, most of these studies have focused on the overall effects of TCC on mental health and cognitive abilities, without delving deeply into the specific neural mechanisms underlying its impact on mental fatigue. Recent research by Qi et al. (2019) has shown that mental fatigue is not only associated with a decline in cognitive performance but also with changes in neural activity patterns (Tran et al., 2020). To gain a deeper understanding of the underlying mechanisms on mental fatigue, researchers have turned to neuroimaging techniques, such as electroencephalography (EEG). EEG provides valuable insights into the electrical activity of the brain, allowing researchers to examine changes in brain function associated with mental fatigue. A meta-analysis found significant differences in brain activity under mental fatigue conditions, revealing increased theta band activity in the brain under mental fatigue conditions (Tran et al., 2020). In a study by He and Hu (2022), TCC was shown to increase EEG alpha wave activity, indicative of a relaxed state, implying a potential mechanism for its fatigue-alleviating effects. Similarly, a study by Pan et al. (2018) examined the effects of TCC intervention on EEG measures in elderly adults. The findings demonstrated that participants who engaged in a single session of TCC exhibited increased alpha power and decreased beta power, indicating a shift toward a more relaxed and attentive state. In fact, the neural mechanisms underlying the alleviation of mental fatigue by TCC remain unclear. Most of these scholars have merely discussed the issue from the perspective of EEG spectrum. In fact, one crucial aspect of cognitive function that is significantly affected by mental fatigue is inhibitory control. Inhibitory control refers to the ability to suppress inappropriate or irrelevant responses, which is essential for successful task performance (Falkenstein, 2006). The Go/NoGo task is a widely used experimental paradigm to assess inhibitory control (Falkenstein et al., 1999). Although previous research has explored the effects of TCC on mental health and cognitive functions by EEG spectrum, no study has yet investigated the mechanisms of TCC in counteracting mental fatigue from the perspective of inhibitory control using the Go/NoGo task. EEG spectrum measures provide a general overview of brain rhythms electrical activity, while event- related potentials (ERPs) are time-locked to specific events or stimuli, allowing for a more precise examination of the neural processes underlying cognitive functions (Sokhadze et al., 2017). For example, the P3 component, in the Go/NoGo task used in this study, is closely associated with the allocation of attentional resources and the evaluation of task-relevant stimuli (Kato et al., 2009). In the context of mental fatigue, changes in the P3 amplitude can indicate how well an individual can still allocate resources for stimulus detection (Kato et al., 2009). The N2 component, especially the NoGo-N2, is considered to be closely related to response inhibition and conflict monitoring (Groom and Cragg, 2015). Mental fatigue may disrupt the normal functioning of these processes, leading to changes in the N2 amplitude. By analyzing the N2 amplitude, we can gain insights into how mental fatigue affects the brain’s ability to inhibit inappropriate responses and monitor conflicts. In this study, these ERP components can provide unique information about the specific neural mechanisms through which TCC may alleviate mental fatigue and improve cognitive control. This study aims to fill this gap in the literature. By examining the ERPs based on the Go/NoGo task, we can gain a more in-depth understanding of how TCC affects the neural processes related to inhibitory control during mental fatigue.

Based on previous research on TCC and cognitive function, as well as the known effects of mental fatigue on cognitive processes, we propose the following hypotheses: (1) After mental fatigue induction, the TCC group will have a greater reduction in subjective mental fatigue (VAS scores) and more significant improvement in reaction times in the Go/NoGo task compared to the aerobic exercise and control groups, indicating better cognitive control. (2) Regarding ERP components, after TCC intervention, the TCC group will show a more significant increase in the P3 amplitude and a more significant decrease in the NoGo-N2 amplitude compared to the other two groups, suggesting enhanced attentional resource allocation and better regulation of response inhibition processes affected by mental fatigue.

This research not only contributes to a more comprehensive understanding of the neural mechanisms by which TCC alleviates mental fatigue in college students but also provides valuable insights for the application of TCC in alleviating mental fatigue. It has the potential to offer new perspectives for the development of non-pharmacological interventions for mental fatigue, which is of great significance in both academic research and practical applications.

## Methods

### Participants and procedure

#### Participants

This study recruited a total of 55 participants. However, during the course of the experiment, three participants were unable to complete the study, resulting in a final sample of 52 participants.

A total of 52 college students specializing in martial arts set direction were recruited. They had completed a minimum of one semester, equivalent to 32 h and demonstrated proficiency in executing the 24-form Yang-style TCC protocol. Furthermore, they exhibited the capability to execute all elements of the TCC protocol to a satisfactory standard. The participants consisted of 28 males and 24 females, as outlined in Table 1. Fifty-two students for this study were recruited from the university, which were randomly assigned into Tai Chi Chuan (TCC, *n* = 18), aerobic exercise (AE, *n* = 17), and control (CON, *n* = 17) groups based on intervention types. Statistical analysis revealed no significant differences between the groups in terms of age, height, weight, BMI, Visual Analog Scale (VAS) (Table 1). Inclusion criteria will include: (1) healthy adults aged between 18 and 30 years with no history of neurological or psychiatric disorders. (2) Participants had a minimum of one semester experience with TCC practice. Exclusion criteria were defined as follows: (1) Chronic illnesses or a history of major illnesses. (2) Experiences of sports-related injuries or fractures within the past 3 years. (3) Engagement in high-intensity physical activities (e.g., basketball, running, skiing, etc.) within 48 h preceding the test. All participants provided written informed consent and understood the experimental process and purpose. This study was reviewed and approved by the Ethics Committee of the University.

**Table 1:** Demographic information and baseline test results of subjects (M ± SD).

	TCC (*n* = 18)	AE (*n* = 17)	CON (*n* = 17)	*F/x^2^*	*p*
Male/female	10/8	9/8	9/8	0.028	0.986
Age (years)	19.82 ± 1.31	20.07 ± 1.15	20.53 ± 1.58	1.207	0.307
Height (cm)	173.44 ± 4.75	172.18 ± 6.22	171.82 ± 5.78	0.408	0.666
Body mass (kg)	67.29 ± 7.88	68.41 ± 7.33	65.94 ± 9.28	0.386	0.681
BMI (kg/m^2^)	22.30 ± 1.72	23.01 ± 1.03	22.25 ± 2.19	1.004	0.373
Education (years)	14.72 ± 1.03	14.53 ± 0.94	15.12 ± 1.41	1.143	0.327
TCC Practice frequency (times/week)	3.39 ± 0.53	3.58 ± 0.90	3.62 ± 0.75	0.472	0.626
Duration (min/session)	64.44 ± 13.81	68.53 ± 11.83	62.94 ± 9.20	1.021	0.367
VAS	26.50 ± 7.64	26.18 ± 7.02	25.18 ± 7.01	0.157	0.854

#### Procedure

This study employed a randomized controlled design. Three groups of participants were induced into mental fatigue using the Stroop task, after which the TCC and AE groups engaged in 20 min of TCC practice or ergo-cycle exercise, respectively, while the CON group participated in a sitting rest for 20 min. Subjective levels of fatigue and cognitive performance were assessed using Visual Analog Scale (VAS) and Go/NoGo task before and after mental fatigue. Additionally, EEG signals of participants were collected for the Go/NoGo task.

### Intervention program

#### Tai Chi Chuan exercise

Participants in the TCC group conducted 24-form simplified Yang-style TCC exercises for 20 min. In this protocol, participants executed a sequence consisting of three repetitions of the 24-form TCC routine (Duan et al., 2024). Exercise intensity was based on their Rating of Perceived Exertion (RPE) of Borg 6–20 scale. All participants reported their RPE scale 11–13, reaching a ‘moderate’ effort level. This aligns with the concept of effort perception, described as the subjective experience of difficulty associated with a physical task (Marcora et al., 2009).

#### Ergo-cycle exercise

The AE group performed aerobic exercise on an ergometer. The exercise intensity was personalized based on the RPE. Participants were asked to maintain an RPE of 12–13 on the Borg 6–20 RPE scale during the 20 - min exercise session (Jacquet et al., 2021). The ergometer was adjusted to ensure a consistent and appropriate workload for each participant. The following Table 2 presented a comparison of the TCC and Ergo-cycle exercises.

**Table 2:** A comparison of the TCC and Ergo-cycle exercises.

Exercise	Tai Chi Chuan	Ergo-cycle
Type	Mind–body exercise	Aerobic exercise
Duration	20 min	20 min
Intensity (Borg scale)	RPE:11–13	RPE: 12–13
Movement pattern	Yang-style TCC	Pedaling on ergometer

### Measures

Before the start of the experiment, a baseline assessment was conducted. Firstly, the subjects’ VAS scores and cognitive performance were collected, followed by a cognitive evaluation by the Go/NoGo task.

#### Mental fatigue task

Numerous previous studies (Catala et al., 2021; Niu et al., 2024; Meymandi et al., 2023; Veness et al., 2017) have established the Stroop task as a reliable experimental model for inducing mental fatigue. It engages participants in a conflict-ridden cognitive process where they must inhibit automatic responses, leading to mental fatigue over time. The 30-min duration used in this study was based on the successful induction of mental fatigue in similar previous research (Catala et al., 2021; Niu et al., 2024; Meymandi et al., 2023; Veness et al., 2017). According to the study of Niu et al. (2024), the implementation of the Stroop task in our study adhered to the following protocol: (1) Utilizing the E-Prime 3.0 software, the task displayed four Chinese characters—"红/red,” “蓝/blue,” “绿/green,” and “黄/yellow”—against a black computer screen backdrop in a sequential manner. Each character was designed to be incongruent, with the displayed color contradicting the character’s semantic meaning (e.g., the character for “绿/green” displayed in a non-green color). (2) Each character was displayed for duration of 1,000 ms, followed by a 500 ms inter-stimulus interval, resulting in a new character presentation every 1,500 ms. (3) Over the course of 30 min, participants were tasked with indicating the color of the presented character by pressing a corresponding key on the keyboard with their right index finger (H for red, J for yellow, K for blue, L for green), prioritizing the character’s color over its meaning and aiming for swift responses. (4) In instances of incorrect responses or failures to respond within the allotted time, the subsequent character was displayed immediately with a yellow text alert. Conversely, timely correct responses were met with blue text feedback, intended to motivate participants to perform the task with both haste and precision.

#### Subjective mental fatigue evaluation

Participants’ subjective levels of mental fatigue were assessed utilizing a VAS specifically designed for mental fatigue, as referenced in prior research (Smith et al., 2019). This VAS consisted of a 100-mm line with endpoints labeled at 0 mm as “No mental fatigue” and 100 mm as “Maximum mental fatigue,” allowing participants to indicate their perceived fatigue by marking a point along the line.

Participants were tasked with rating their mental fatigue on a 10 cm line, with one extremity signifying “no mental fatigue” and the opposite end indicating “utter mental exhaustion.” They marked their subjective state along this line. The VAS was administered at two junctures: initially as a baseline, after Stroop task and subsequently after 20 min of intervention. Participants were allotted 1 min to complete the VAS. A score surpassing 50 indicated the attainment of significant mental fatigue, as delineated in a study (Meymandi et al., 2023).

#### The Go/No-go task

The Go/No-go paradigm is a widely used experimental task designed to assess inhibitory control. Stimuli presentation was controlled by using eprime 3.0, presented on a 17-inch LCD monitor. The stimuli consisted of a series of visual targets (e.g., geometric shapes) that appeared one at a time in the center of the screen. Each target was presented for 500 ms followed by a blank inter-stimulus interval (ISI) of 500 ms.

Participants were seated approximately 70 cm away from the computer screen in a quiet, dimly lit room. They were instructed to respond as quickly and accurately as possible to the go stimuli (e.g., the left arrow) by pressing a designated button on a response pad. In contrast, they were instructed to withhold their response to no-go stimuli (e.g., the right arrow). The Go and No-go stimuli were presented in a randomized order, ensuring that the sequence did not repeat more than twice consecutively.

The task consisted of two blocks, each containing 100 trials (50% Go trials and 50% No-Go trials) (Guo et al., 2015). Before starting the main task, participants underwent a practice session consisting of 20 trials to familiarize themselves with the task requirements. During the practice session, feedback on accuracy and reaction time was provided to ensure understanding of the instructions. No feedback was given during the actual task.

Data were analyzed by using eprime 3.0. Reaction times (RTs) was recorded for Go trials. RTs for correct go responses were averaged for each participant.

#### EEG recording, preprocessing and ERPs

EEG data were captured utilizing a mobile EEG device (eego™mylab, ANT Neuro, Netherlands) equipped with a 64-channel cap adhered to the international 10–10 system guidelines. In preparation for the study, participants underwent a hair wash to diminish the impact of scalp oils and reduce electrode impedance. Optimal contact between the electrodes and scalp was facilitated by applying conductive gel to each electrode location, ensuring that impedance levels were maintained below 10 KΩ. The EEG recordings were conducted at a sampling rate of 1,000 Hz, with a bandpass filter set between 0.1 and 50 Hz. The ground electrode was situated between the FPz and Fz channels, while the CPz electrode functioned as the reference.

The EEG data underwent a series of preprocessing steps to enhance signal quality (Niu et al., 2024): (1) a bandpass filter was applied to retain frequencies within the range of 0.5–30 Hz. (2) A 50 Hz notch filter was utilized to eliminate power line interference. (3) The sampling rate was reduced to 250 Hz to decrease data redundancy. (4) Interpolation was employed to correct for channels with excessive noise. (5) The data were re-referenced to the average signal of the bilateral mastoids. (6) Independent Component Analysis (ICA) was conducted to identify and remove components related to ocular, muscular, and cardiac activities. (7) The EEG data were segmented into epochs spanning from −200 to 600 ms for further analysis.

ERPs were computed by averaging the segmented EEG data for each condition (Go and No-go) separately. The P3 and N2 components were identified visually by inspecting the grand-average ERPs. The peak latency and amplitude of the N2 (200–350 ms) and P3 (250–500 ms) were determined by identifying the most negative or positive deflection within above time window at Fz, Cz and Pz (Guo et al., 2015).

### Statistical analysis

For the sample size estimation, based on a 3 × 3 repeated measures ANOVA design, the effect size (Cohen’s *f*) was set at 0.25, with a significance level of 0.05 and statistical power of 0.9. *A priori* estimated total sample size was 45 participants. Statistical analysis of the data and figures was conducted using SPSS 20.0 and GraphPad Prism 9.0. In this study, demographic data were analyzed using one-way ANOVA. VAS, RTs, P3 and N2 amplitude, and P3 and N2 latencies, were tested by 3 × 3 two-way repeated measures ANOVA. The between-subjects variable was group (TCC, AE, and CON), and the within-subject variable was the test time (Pre, Post-0, Post-20). *Post-hoc* multiple comparisons were corrected using the Tukey method for *p*-values. Effect sizes were also reported: Cohen’s d was used for pairwise comparisons, and η_p_^2^ for ANOVA analyses. The significance level was set at *α* = 0.05. In this study, all values conform to a normal distribution, which were expressed as mean ± standard deviation (X ± SD).

## Results

### VAS

In the present study, significant time × group interaction effects on the VAS scores were observed (*F* (4, 98) = 6.079, *p* = 0.0002, η_p_^2^ = 0.198), indicating differential changes in VAS scores over time among the TCC, AE, and CON groups. Significant main effects of time (*F* (2, 98) = 174.6, *p* < 0.0001, η_p_^2^ = 0.781) and group (*F* (2, 49) = 4.334, *p* = 0.0185, η_p_^2^ = 0.194) were also noted.

**Figure 1:**
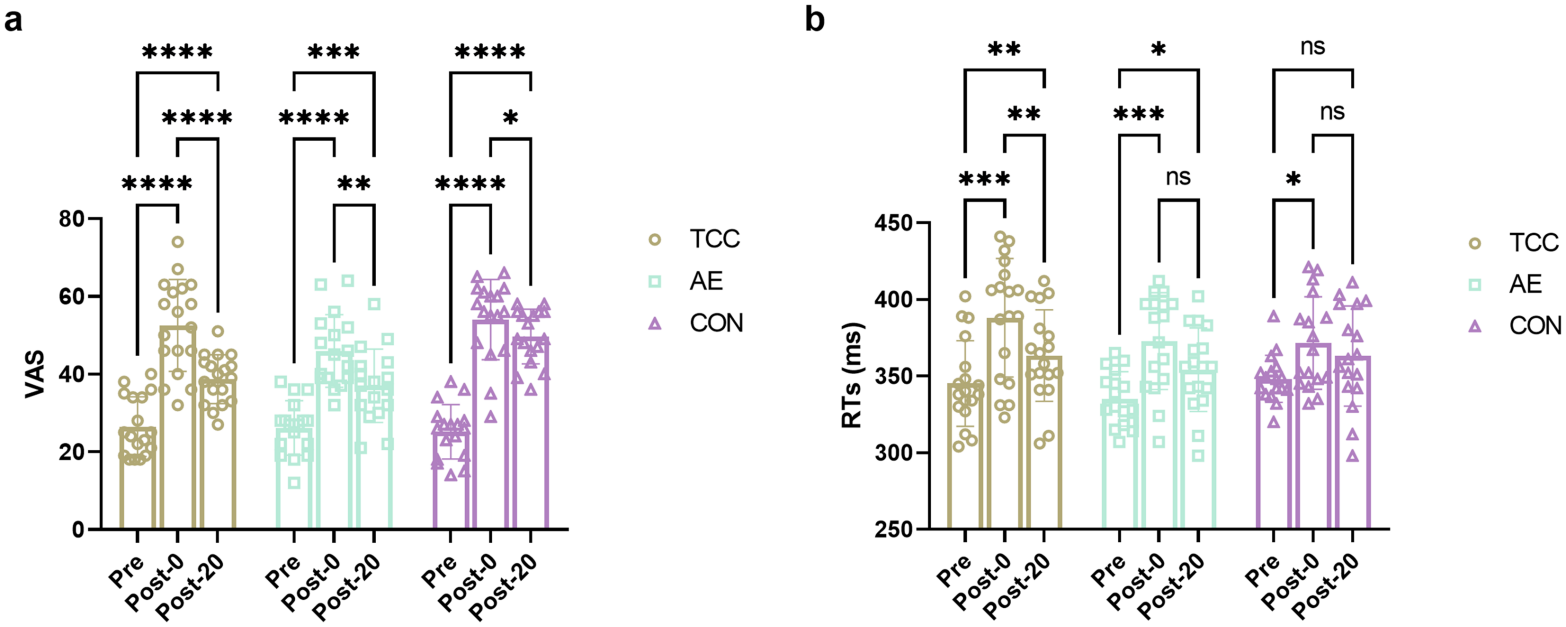
Changes and comparisons in VAS **(a)** and RTs **(b)** of subjects in different groups performing the Go/NoGo task at different time points (Pre, Post-0, Post-20). *, **, *** represent *p* < 0.05, *p* < 0.01, *p* < 0.001, respectively; ns indicates no significant difference (*p* > 0.05).

For the TCC group, a significant effect of time on VAS scores was detected (*F* (2, 34) = 54.66, *p* < 0.0001, η_p_^2^ = 0.762), with *post-hoc* analyses revealing a significant increase in VAS scores from Pre to Post-0 (*t* = 11.52, *p* < 0.0001, *d* = 1.92), followed by a significant decrease at Post-20 after TCC exercise (*t* = 9.609, *p* < 0.0001, *d* = 1.60). The AE group also showed a significant effect of time on VAS scores (*F* (2, 32) = 38.48, *p* < 0.0001, η_p_^2^ = 0.706), with a significant increase from Pre to Post-0 (*t* = 11.30, *p* < 0.0001, *d* = 1.94), and a subsequent significant decrease at Post-20 after exercise (*t* = 5.822, *p* = 0.0003, *d* = 1.00). The CON group exhibited a significant effect of time on VAS scores as well (*F* (2, 32) = 97.88, *p* < 0.0001, η_p_^2^ = 0.859), with a significant increase from Pre to Post-0 (*t* = 14.84, *p* < 0.0001, *d* = 2.55), and a significant decrease at Post-20 after a 20-min rest (*t* = 3.651, *p* = 0.05, *d* = 0.626) (Figure 1a).

At the Pre stage, there was no significant effect of group on VAS scores (*F* (2, 49) = 0.1573, *p* = 0.8549), indicating no differences among the three groups. Similarly, at Post-0, there was no significant effect of group (*F* (2, 49) = 2.846, *p* = 0.0677), with no differences among the groups. However, at Post-20, a significant effect of group was observed (*F* (2, 49) = 13.73, *p* < 0.0001, η_p_^2^ = 0.359). *Post-hoc* analyses indicated that both the TCC (*t* = 5.968, *p* = 0.0003, *d* = 1.64) and AE (*t* = 6.812, *p* < 0.0001, *d* = 1.52) groups had significantly lower VAS scores than the CON group, but there were no significant differences between the TCC and AE groups (Figure 1a). These findings suggest that both TCC and AE are effective in alleviating mental fatigue, with effects superior to those of passive rest.

### RTs

In the analysis of RTs, no significant time × group interaction was found (*F* (4, 98) = 1.069, *p* = 0.376). However, a significant main effect of time was observed on RTs (*F* (2, 98) = 36.53, *p* < 0.0001, η_p_^2^ = 0.425). No significant effect of group was detected (*F* (2, 49) = 1.036, *p* = 0.3627).

Within-group analyses revealed significant effects of time on RTs for the TCC group (*F* (2, 34) = 20.53, *p* < 0.0001, η_p_^2^ = 0.547), with *post-hoc* comparisons indicating significant improvements from Pre to Post-0 (*t* = 7.744, *p* = 0.0001, *d* = 1.29) and from Post-0 to Post-20 (*t* = 5.162, *p* = 0.0053, *d* = 0.861). The AE group also showed significant effects of time on RTs (*F* (2, 32) = 14.17, *p* < 0.0001, η_p_^2^ = 0.469), with significant improvements from Pre to Post-0 (*t* = 7.28, *p* = 0.0003, *d* = 1.24) and a trend toward significance from Post-0 to Post-20 (*t* = 3.533, *p* = 0.05, *d* = 0.61). The CON group demonstrated a significant effect of time on RTs (*F* (2, 32) = 5.053, *p* = 0.0124, η_p_^2^ = 0.834), with significant improvements from Pre to Post-0 (*t* = 3.945, *p* = 0.033, *d* = 0.966) and no significant change from Post-0 to Post-20 (*p* = 0.182) (Figure 1b).

These findings suggest that both the TCC and AE interventions led to significant improvements in RTs compared to the CON group, with the TCC group showing more pronounced effects.

### P3 component

**Figure 2:**
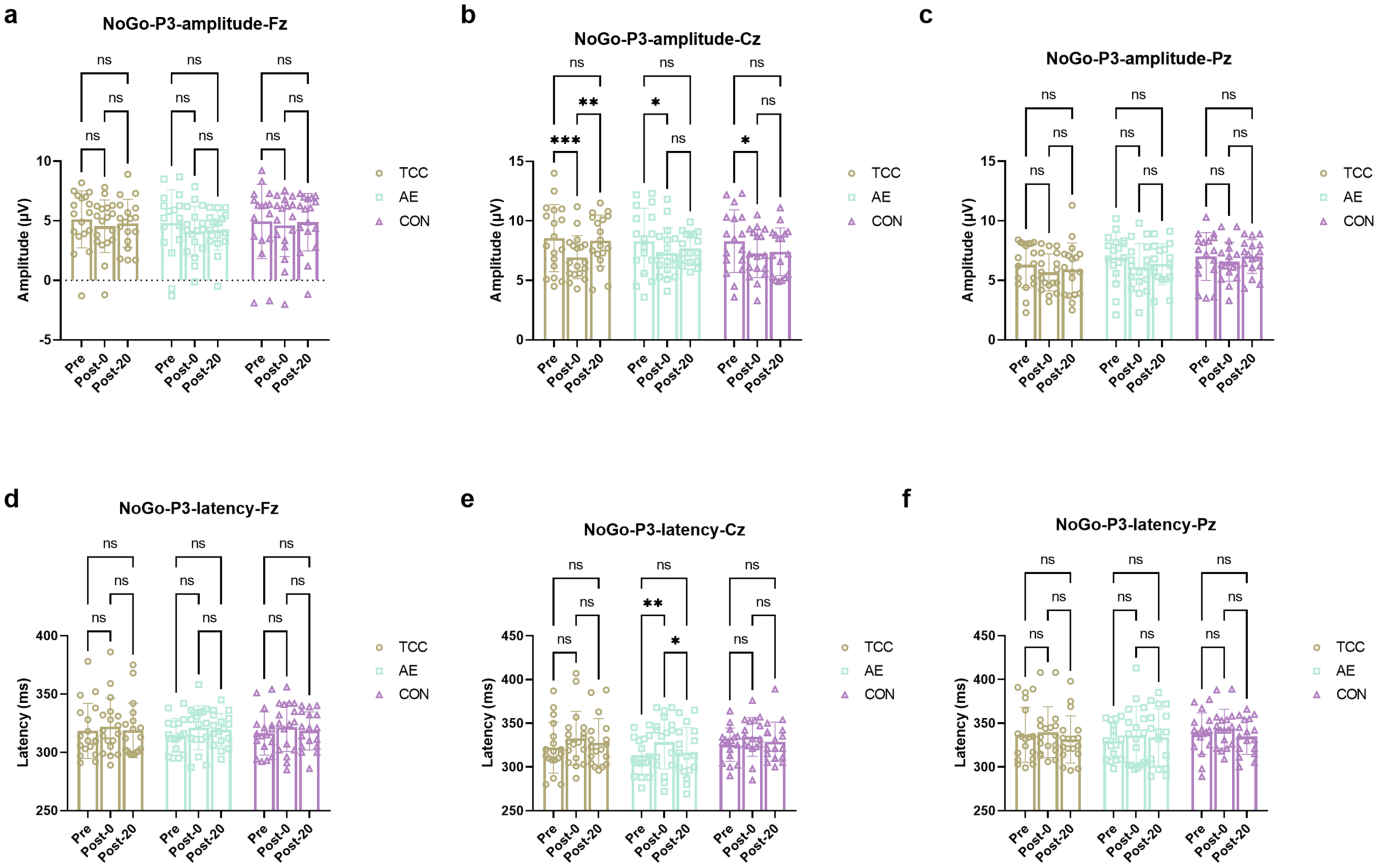
Changes and comparisons in NoGo-N2 amplitudes at different electrode sites Fz **(a)**, Cz **(b)**, Pz **(c)** and NoGo-N2 latencies at Fz **(d)**, Cz **(e)**, Pz **(f)** sites for subjects in different groups performing the Go/NoGo task at different time points (Pre, Post-0, Post-20). *, ** represent *p* < 0.05, *p* < 0.01, respectively; ns indicates no significant difference (*p* > 0.05).

Despite previous research on the positive effects of exercise on mental health and cognition, there is a lack of studies exploring the neural mechanisms of TCC in alleviating mental fatigue from the perspective of ERP components related to inhibitory control. In the analysis of NoGo-P3 amplitudes across three groups and test phases at the Fz, Cz, and Pz sites, significant differences were observed only at the Cz site. At this site, no significant time × group interaction was found (*F* (4, 98) = 1.486, *p* = 0.2123), but a significant effect of time was present (*F* (2, 98) = 13.38, *p* < 0.0001, η_p_^2^ = 0.214), with no significant effect of group (*F* (2, 49) = 0.08017, *p* = 0.9231). For the TCC group, a repeated-measures ANOVA revealed a significant time effect on NoGo-P3 amplitude (*F* (2, 34) = 5.191, *p* = 0.01, η_p_^2^ = 0.235). *Post-hoc* comparisons indicated that the amplitude at Post-0 was significantly lower than at Pre (*t* = 5.587, *p* = 0.0028, *d* = 0.939), and the amplitude at Post-20 was significantly higher than at Post-0 (*t* = 4.227, *p* = 0.0213, *d* = 0.705). The AE group showed a significant time effect (*F* (2, 32) = 4.381, *p* = 0.0208, η_p_^2^ = 0.215), with a significant decrease from Pre to Post-0 (*t* = 4.194, *p* = 0.0234, *d* = 0.722), and no significant change from Post-0 to Post-20. The CON group also exhibited a significant time effect (*F* (2, 32) = 7.195, *p* = 0.0026, η_p_^2^ = 0.310), with a significant decrease from Pre to Post-0 (*t* = 3.995, *p* = 0.031, *d* = 0.685), and no significant change from Post-0 to Post-20. These results suggest that while all groups experienced a decrease in NoGo-P3 amplitude immediately following the intervention, only the TCC group showed a significant recovery by Post-20, highlighting the potential benefits of TCC in modulating cognitive recovery post-mental fatigue (Figure 2).

**Figure 3:**
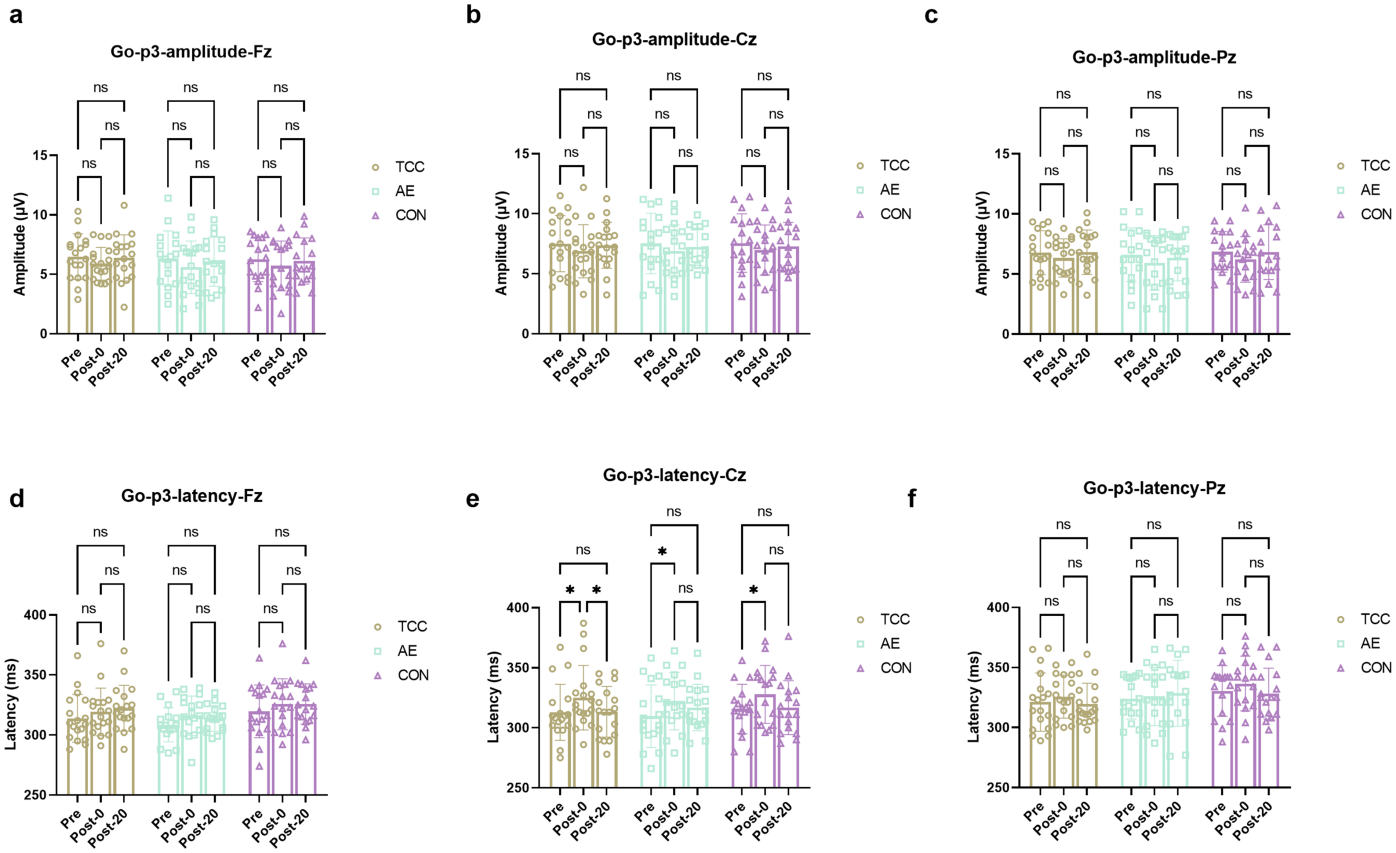
Changes and comparisons in NoGo-P3 amplitudes at different electrode sites Fz **(a)**, Cz **(b)**, Pz **(c)** and NoGo-P3 latencies at Fz **(d)**, Cz **(e)**, Pz **(f)** for subjects in different groups performing the Go/NoGo task at different time points (Pre, Post-0, Post-20). *, ** represent *p* < 0.05, *p* < 0.01; ns indicates no significant difference (*p* > 0.05).

For the Go-P3 amplitudes, there were no effects of time and group, as well as their interaction were not significant. However, there was a significant effect of time on the Go-p3 latencies at the Cz site (*F* (2, 98) = 10.09, *p* = 0.0001, η_p_^2^ = 0.171), with a significant delay from Pre to Post-0 regardless of TCC (*t* = 3.553, *p* = 0.036, *d* = 0.592), AE (*t* = 3.557, *p* = 0.0358, *d* = 0.61) or CON (*t* = 3.424, *p* = 0.045, *d* = 0.587) groups. And only in the TCC group the Go-p3 latencies significantly shortened from the Post-0 to Post-20 (*t* = 3.408, *p* = 0.0465, *d* = 0.567) (Figure 3). This is a novel finding that was not fully explored in previous studies. It reveals the unique role of TCC in improving the cognitive processing speed after mental fatigue, providing new insights into the regulatory mechanism of TCC on brain activity.

### N2 component

**Figure 4:**
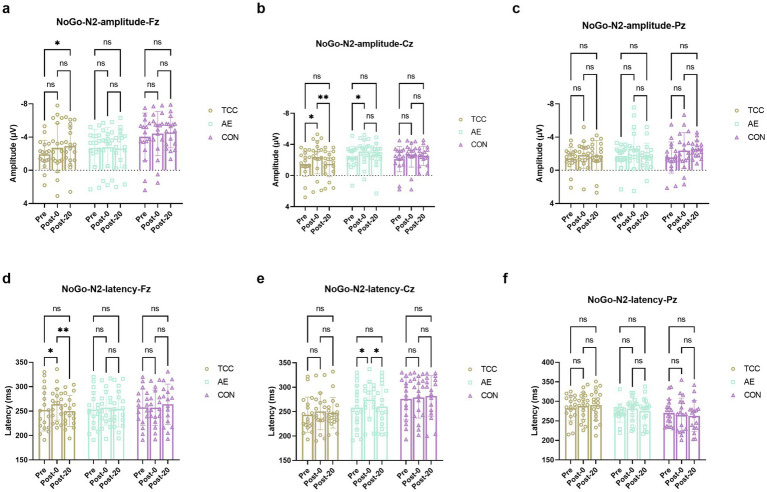
Changes and comparisons in Go-N2 amplitudes at different electrode sites Fz **(a)**, Cz **(b)**, Pz **(c)** and Go-N2 latencies at Fz **(d)**, Cz **(e)**, Pz **(f)** sites for subjects in different groups performing the Go/NoGo task at different time points (Pre, Post-0, Post-20). * indicates *p* < 0.05; ns indicates no significant difference (*p* > 0.05).

For the NoGo-N2 amplitudes at the Cz, there were effects of time (*F* (2, 98) = 9.471, *p* = 0.0002, η_p_^2^ = 0.162), but no main effects of condition and interaction. For the TCC, Pre vs. Post-0: −1.51 ± 1.63 vs. −2.37 ± 2.15 μV (*t* = 4.160, *p* = 0.0113, *d* = 0.694); Post-0 vs. Post-20: −2.37 ± 2.15 vs. −1.49 ± 1.64 μV (*t* = 4.251, *p* = 0.0093, *d* = 0.709). For the AE, Pre vs. Post-0: −2.34 ± 1.38 vs. −3.11 ± 1.52 μV (*t* = 3.569, *p* = 0.035, *d* = 0.612) (Figure 4). For the NoGo-N2 amplitudes at the Fz and Pz sites, there were no effects of time and condition, as well as their interaction were not significant.

For the NoGo-N2 latencies at the Fz site, there was a significant time × group interaction (*F* (4, 98) = 2.586, *p* = 0.0416, η_p_^2^ = 0.095). Further *post-hoc* pairwise comparisons demonstrated that only in the TCC group there was a significant effect of time on the NoGo-N2 latencies. For the TCC, Pre vs. Post-0: 252.4 ± 43.1 vs. 264.1 ± 32.6 ms (*t* = 3.628, *p* = 0.0315, *d* = 0.604); Post-0 vs. Post-20: 264.1 ± 32.6 vs. 250.3 ± 31.7 ms (*t* = 4.302, *p* = 0.0084, *d* = 0.715) (Figure 4). However, there were no main effects of time and condition or interactions at Cz and Pz sites.

**Figure 5:**
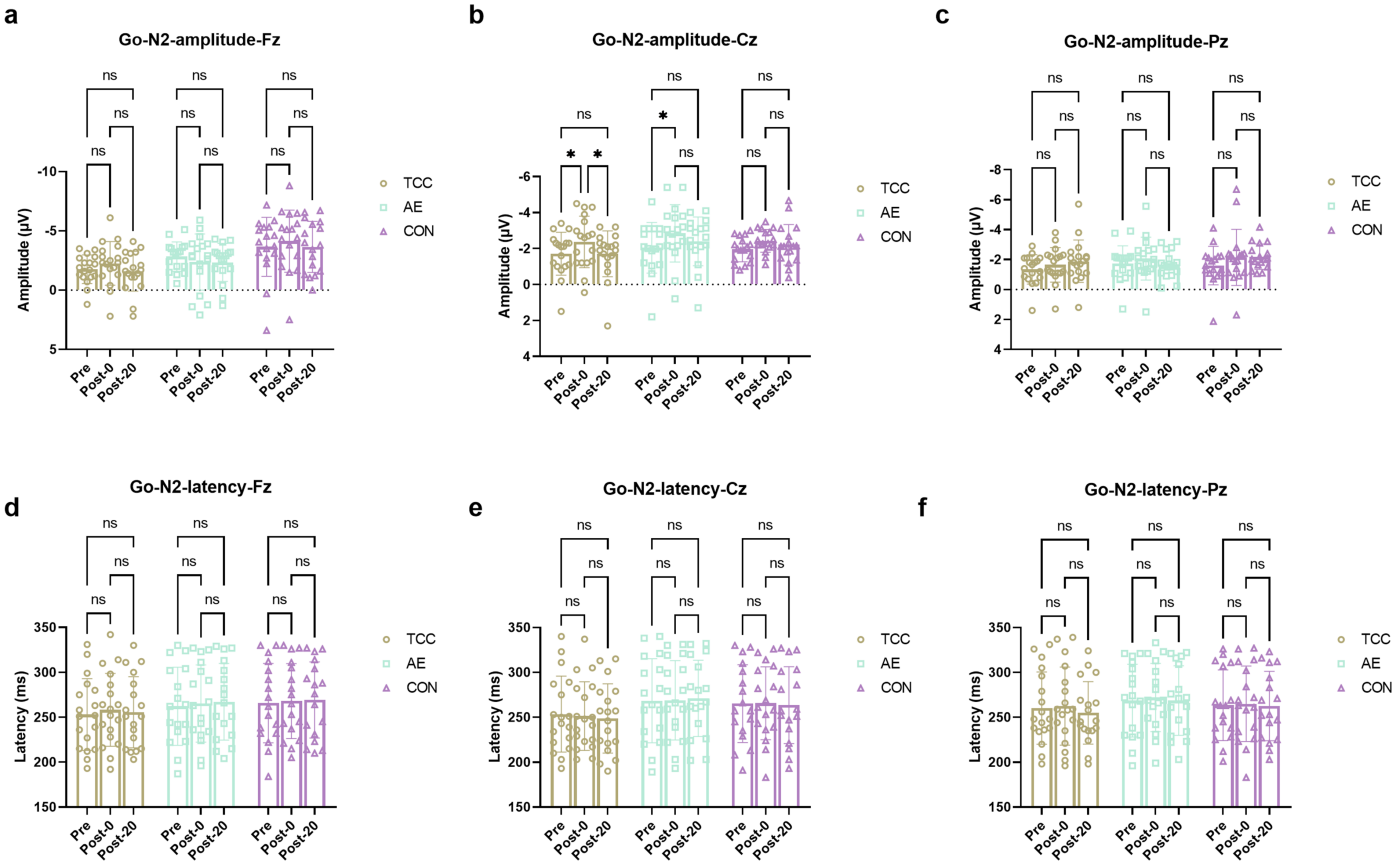
Changes and comparisons in Go-P3 amplitudes at different electrode sites Fz **(a)**, Cz **(b)**, Pz **(c)** and Go-P3 latencies at Fz **(d)**, Cz **(e)**, Pz **(f)** sites for subjects in different groups performing the Go/NoGo task at different time points (Pre, Post-0, Post-20). * indicates *p* < 0.05, *p* < 0.01; ns indicates no significant difference (*p* > 0.05).

For the Go-N2 amplitudes at the Cz, there were effects of time (*F* (2, 98) = 8.892, *p* = 0.0003, η_p_^2^ = 0.153), but no main effects of group and interaction. For the TCC group, Pre vs. Post-0: −1.71 ± 0.98 vs. −2.37 ± 1.16 μV (*t* = 3.498, *p* = 0.0397, *d* = 0.584); Post-0 vs. Post-20: −2.37 ± 1.16 vs. −1.71 ± 1.41 μV (*t* = 3.504, *p* = 0.0393, *d* = 0.586). For the AE group, Pre vs. Post-0: −2.05 ± 1.18 vs. −2.85 ± 1.43 μV (*t* = 4.149, *p* = 0.0115, *d* = 0.712) (Figure 5). For the Fz and Pz sites, there were no effects of time and condition, as well as their interaction were not significant. For the Go-N2 latencies, there were no main effects of time and condition or interactions at the Fz, Cz, and Pz sites.

These results further demonstrate the regulatory effect of TCC on the conflict monitoring process during mental fatigue, which is an important part of inhibitory control.

## Discussion

In this study, all groups demonstrated evidence of mental fatigue following a 30-min fatigue-inducing task, as evidenced by increased VAS scores and prolonged reaction times. This finding suggests that the task was effective in inducing mental fatigue. Furthermore, following the induction of mental fatigue, the amplitudes of the NoGo-P3 and NoGo-N2 components were significantly reduced, and their latencies were significantly prolonged during the Go/NoGo task, with the most pronounced effects observed at the Cz electrode site. After a 20-min intervention, TCC practice led to a more substantial improvement in these indices compared to both aerobic exercise and a control group.

In this study, the decline in behavioral performance (slower RTs) after mental fatigue, which was in line with a study of (Junior et al., 2020), may primarily be associated with a reduction in the allocation of attentional resources due to cognitive fatigue (Holtzer et al., 2011). Kok (1997) have posited that the availability of attentional resources plays a decisive role in cognitive performance, with a scarcity of such resources leading to poorer behavioral outcomes.

Previous neurophysiological research has shown that the P3 and N2 components of ERPs are closely related to cognitive processes such as attentional resource allocation, response inhibition, and conflict monitoring (Kok, 1997; Dimoska et al., 2006). Therefore, changes in these components can be used as reliable indicators to evaluate the impact of mental fatigue and interventions on cognitive function.

Go-P3 is a component of ERPs elicited by the Go/NoGo task. The Go-P3 component typically emerges around 300 milliseconds after stimulus onset, predominantly distributed over the central-parietal region, with the largest amplitude near the midline (Dimoska et al., 2006). It reflects the processing and evaluation of task-relevant stimuli (Guo et al., 2015). In our study, it was observed that following the fatigue-inducing task, the amplitude of the Go-P3 significantly decreased, suggesting that participants found it increasingly difficult to allocate resources for stimulus detection during task execution. These results are in close alignment with those of Guo et al. (2015), who found that cognitive fatigue significantly reduced the amplitude of Go-P3 at the Cz site. However, our findings differ from those of Kato et al. (2009), who reported no effect of cognitive fatigue on the amplitude of Go-P3. This discrepancy may be attributed to the different ratios of Go to NoGo trials used in the studies (Kato et al., 2009). In their research, the probability of Go trial stimuli (80%) was greater than that of NoGo trial stimuli (20%). Cognitive fatigue may lead to a decrease in the availability of cognitive resources (Boksem et al., 2006; Kato et al., 2012; Guo et al., 2015), and to maintain rapid responses, the most reasonable strategy would be to allocate limited resources to Go stimuli rather than NoGo stimuli. Consequently, due to the effects of cognitive fatigue, participants may not be able to allocate sufficient attentional resources specifically to NoGo stimuli, instead directing more attentional resources to Go stimuli, ultimately resulting in no change in the amplitude of Go-P3 (Guo et al., 2015). In our study, the probability of Go and NoGo trial stimuli was 50% each, thus requiring participants to detect both types of stimuli with equal probability and to allocate an equal amount of attentional resources to both Go and NoGo stimuli. In this study, following mental fatigue, TCC intervention led to a significant increase in the amplitudes of Go-P3 in participants, whereas aerobic exercise and sitting interventions did not result in a significant increase. These findings suggest that practicing TCC after mental fatigue plays an important role in alleviating the decline in cognitive abilities caused by psychological fatigue.

In the Go/NoGo task, the NoGo-P3 emerges between 300 and 500 ms after stimulus onset, primarily distributed in the prefrontal cortex and anterior cingulate gyrus (Guo et al., 2015). It is an indicator of the late stage of inhibitory processing and may reflect inhibitory processes in or around the late motor or premotor cortices (Dimoska et al., 2006). This study found that the amplitude of NoGo-P3 at the Cz site significantly decreased after the fatigue task, suggesting that subjects’ response inhibition was impaired after mental fatigue (Dimoska et al., 2006). In agreement with the Kato’s research (Kato et al., 2009), they also found that the amplitude of NoGo-P3 decreased with the progression of the fatigue task, indicating that mental fatigue affected the controlled processing of response inhibition. Although we required the participants to respond as quickly and accurately as possible when completing the Go/NoGo task in order to avoid the influence of the response strategy on P3, there might still be some changes in the response strategy during the task execution, which could have an impact on the amplitude and latency of P3 (Doucet and Stelmack, 1999).

In this study, another ERP component N2 also changed. In the Go/NoGo task, the amplitude of N2 is usually associated with conflict monitoring (Jodo and Kayama, 1992; Falkenstein et al., 1999). Studies have shown that the N2 component under the NoGo task has a larger negative amplitude relative to the Go task, which is known as the NoGo-N2 effect and is considered closely related to response inhibition (Jodo and Kayama, 1992; Falkenstein et al., 1999). The NoGo-N2 is mainly distributed in the frontocentral area, appearing 200–300 ms after the NoGo stimulus, reflecting a top-down response inhibition mechanism, and some studies suggest that it is related to early conflict monitoring in the inhibition process (Falkenstein, 2006). In this study, the amplitude of NoGo-N2 increased after mental fatigue. The increase in NoGo-N2 amplitude likely represents a compensatory mechanism in the face of declining cognitive control due to mental fatigue. A decline in cognitive control is a more expected outcome of mental fatigue. As mental resources become depleted, the brain attempts to maintain task performance by heightening certain aspects of conflict-related processing, reflected in the elevated NoGo-N2 amplitude (Fan et al., 2019). This is in line with previous research indicating that mental fatigue impairs overall cognitive function (Filtness and Naweed, 2017; Peng et al., 2021), and the increase in NoGo-N2 may be an effort to counteract this impairment. However, in another study, researchers found a trend toward increased amplitude of NoGo-N2 caused by mental fatigue, but no significant difference (Guo et al., 2015). The differences in the results might be related to the fact that the degree of mental fatigue in our study is higher than that in the study by Guo et al. (2015). In this study, after mental fatigue, the subjects’ NoGo-N2 amplitude significantly decreased through TCC practice. We believe this may be due to TCC practice regulating the body’s conflict monitoring process.

Additionally, in this study, we also found that mental fatigue induced an increase in the amplitude of Go-N2. Go-N2 typically appears 200–300 milliseconds after the Go stimulus and is mainly distributed in the frontocentral region (Jodo and Kayama, 1992; Falkenstein et al., 1999). Go-N2 is believed to be related to conflict monitoring and the preparation of motor inhibition, reflecting the preparation and monitoring of an impending response (Jodo and Kayama, 1992; Falkenstein et al., 1999). Studies have shown that as task difficulty increases, the amplitude of Go-N2 is enhanced, which may indicate that under high cognitive load conditions, the brain requires more cognitive resources to handle conflicts, leading to an increase in the amplitude of Go-N2 (Liu et al., 2024). In this study, subjects performed cognitive tasks after mental fatigue, requiring the brain to consume more cognitive resources to complete the cognitive tasks, leading to the increase in Go-N2 amplitude. Additionally, in this study, mental fatigue also induced a significant prolongation of the latency of NoGo-N2 and Go-P3 in subjects. The delay in the latency of NoGo-N2 indicates that mental fatigue affected the process of inhibitory activity, slowing down the speed of response inhibition (Kato et al., 2009). More specifically, mental fatigue weakened the speed of conflict detection between Go and NoGo stimuli.

In this study, aerobic exercise did not produce a significant effect similar to that of TCC. We analyze the reasons as follows. TCC is a mind–body exercise that emphasizes slow, coordinated movements, deep breathing, and mindfulness (Caldwell et al., 2016). These aspects may have a unique impact on the neural processes related to mental fatigue alleviation. For example, the slow, rhythmic movements of TCC could enhance neural plasticity in the prefrontal cortex, which is crucial for cognitive control (Shen et al., 2021; Yao et al., 2021; Yu et al., 2018). In contrast, aerobic exercise mainly targets cardiovascular fitness and may have a more indirect effect on cognitive function. Additionally, it’s also possible that the experimental design factors contributed to the differential effects. The intensity, duration, and frequency of the aerobic exercise in our study may not have been optimal for eliciting the same level of cognitive benefits.

## Conclusion

The current study suggests that practicing TCC may serve as a potential intervention for reducing mental fatigue during the execution of sustained cognitive tasks. Through the practice of TCC, not only was mental fatigue alleviated, but cognitive performance was also improved. The ERP data further indicate that, compared to the aerobic exercise group and the control group, TCC better facilitated attentional control and inhibition in selective reaction tasks following mental fatigue. In summary, these findings demonstrate that practicing TCC could alleviate mental fatigue in college students encountered in performing sustained cognitive tasks at both behavioral and cognitive levels.

However, it is crucial to acknowledge the limitations of this study. The relatively small sample size of 52 college students may limit the generalizability of our results. The VAS scale is widely used to assess subjective fatigue. However, it is a subjective reporting method and is greatly influenced by individual cognition and emotions. It lacks objective physiological index verification. For example, different participants may have different perceptions and scoring criteria for “fatigue,” which may lead to measurement errors. Additionally, in the aerobic exercise group, although the intensity was set according to the RPE, the actual physiological responses of different individuals to the same RPE may vary, which may interfere with the exercise effect. Future research should focus on two key aspects. First, more ecologically valid methods of inducing mental fatigue are needed. Second, expand the scope of exercise forms for comparison. Incorporate activities such as yoga, Pilates, or team sports, to determine which exercise is most effective in combating mental fatigue. This will offer more targeted advice for managing mental fatigue through physical activity.

## Data Availability

The original contributions presented in the study are included in the article/supplementary material, further inquiries can be directed to the corresponding author.
